# A Hybrid Approach for Biomarker Discovery from Microarray Gene Expression Data for Cancer Classification

**Published:** 2007-02-22

**Authors:** Yanxiong Peng, Wenyuan Li, Ying Liu

**Affiliations:** 1 Laboratory for Bioinformatics and Medical Informatics; 2 Department of Computer Science; 3 Department of Molecular and Cell Biology, University of Texas at Dallas, Richardson, TX 75083-0688, U.S.A

**Keywords:** Biomarker discovery, Gene expression, Cancer classification, Microarray, Gene selection

## Abstract

Microarrays allow researchers to monitor the gene expression patterns for tens of thousands of genes across a wide range of cellular responses, phenotype and conditions. Selecting a small subset of discriminate genes from thousands of genes is important for accurate classification of diseases and phenotypes. Many methods have been proposed to find subsets of genes with maximum relevance and minimum redundancy, which can distinguish accurately between samples with different labels. To find the minimum subset of relevant genes is often referred as biomarker discovery. Two main approaches, filter and wrapper techniques, have been applied to biomarker discovery. In this paper, we conducted a comparative study of different biomarker discovery methods, including six filter methods and three wrapper methods. We then proposed a hybrid approach, FR-Wrapper, for biomarker discovery. The aim of this approach is to find an optimum balance between the precision of the biomarker discovery and the computation cost, by taking advantages of both filter method’s efficiency and wrapper method’s high accuracy. Our hybrid approach applies Fisher’s ratio, a simple method easy to understand and implement, to filter out most of the irrelevant genes, then a wrapper method is employed to reduce the redundancy. The performance of FR-Wrapper approach is evaluated over four widely used microarray datasets. Analysis of experimental results reveals that the hybrid approach can achieve the goal of maximum relevance with minimum redundancy.

## Introduction

DNA microarrays, among the most rapidly growing tools for genome analysis, are introducing a paradigmatic change in biology by shifting experimental approaches from single gene studies to genome-level analyses (Blaschke et al. 2001; Liu et al. 2005; Xu et al. 2003). Increasingly accessible microarray platforms allow the rapid generation of large expression data sets. Analysis of these high-throughput data poses both opportunities and challenges to the biologists, statisticians, and computer scientists. One of the most important characteristics in microarray data is the very high dimensionality (large number of features or genes) with a small number of samples. There are over thousands of genes and at most several hundreds of samples in the data set. Such characteristics, which have never existed in any other type of data, have made the traditional data mining and analysis methods not effective, and therefore attracted the focus of recent research. Among these methods, a crucial approach is to select a small portion of informative genes for further analysis, such as disease classification and the discovery of structure of the genetic network (Li et al. 2006). Due to the drastic size difference of genes and samples, the step of gene selection is also the need of solving the well-known problem “curse of dimensionality” in statistics, data mining and machine learning (Donoho, 2000).

However, quite different from the traditional feature selection in other data sets such as text (Yang and Pedersen, 1997), the final goal of gene selection is to discover “biomarkers,” a minimal subset of genes that not only are differentially expressed across different sample classes, but also contains most relevant genes without redundancy. These two characteristics distinguish the task of discovering “biomarker” from the common feature selection tasks.

Recent gene selection methods fall into two categories: filter methods and wrapper methods (Inza et al. 2004). Filter methods select the features by evaluating the goodness of the features based on the intrinsic characteristics, which determines their relevance or discriminant powers with regards to the class labels (Ding and Peng, 2003; Inza et al. 2004; Yu and Liu, 2004). Most existing filter methods follow the methodologies of statistical tests (e.g. *t*-test, *F*-test) (Ding and Peng, 2003; Jaeger et al. 2003) and information theory (e.g. mutual information or information gain) to rank the genes. The common way for gene selection is to choose the top-ranked genes, say top 50 (Golub et al. 1999) or 150 genes (Li et al. 2004). The number of the genes selected is subjectively determined with trial-and-error. Since the gene ranking is based on a univariate scoring metric, the ranking of a gene is computed in isolation from all other genes, or at most in combinations of pairs of genes (Diaz-Uriarte and Alvarex de Andrez, 2006). As a result, the genes selected could be highly correlated among themselves, which raises the issue of “redundancy” of feature set (Diaz-Uriarte and Alvarex de Andrez, 2006; Ding and Peng, 2003). In filter methods, the gene selection is independent of any learning method (e.g. classifier), and therefore, filter methods have better generalization property (Ding and Peng, 2003; Yu and Liu, 2004). In wrapper methods, gene selection is closely “embedded” in the classifier. The goodness and usefulness of a gene subset is evaluated by the estimated accuracy of the classifier, which was trained only with the subset of genes. Wrapper methods can derive a gene subset with a very small number of non-redundant genes (Ding and Peng, 2003). Because the characteristics of the gene subset match that of the classifier, wrapper methods often yield high classification/prediction accuracy. However, wrapper methods are computationally expensive for data sets with large number of features. Therefore, wrapper methods, which are popular in machine learning applications, are not extensively used in microarray data analysis (Chu et al. 2005; Inza et al. 2002). Because of its computational efficiency, filter methods are adopted by most of works in microarray data analysis, but with the cost of having lower prediction accuracy than wrapper methods.

Comparative studies have been conducted to evaluate different feature selection methods for gene selection (Chai and Domeniconi, 2004; Li et al. 2004; Liu et al. 2002). However, there is no evaluation on various biomarker discovery methods. Therefore, in this paper, we first compared and evaluated different gene selection, especially biomarker discovery methods, including filter methods and wrapper methods. Then we proposed a hybrid gene selection approach, FR-Wrapper, for biomarker discovery. The aim of this approach is to find an optimum balance between the precision of the biomarker discovery and the computation cost, by taking advantages of both filter method’s efficiency and wrapper method’s high accuracy. Xing et al. (2001) proposed a hybrid of filter and wrapper methods to gene selection, in which a complicated Markov Blanket filter was applied. Our hybrid approach applies Fisher’s ratio, a simple method easy to understand and implement, to filter out most of the irrelevant genes, then a wrapper method is employed to reduce the redundancy. The performance of FR-Wrapper approach is evaluated over four widely used microarray datasets. Experimental results showed that the hybrid approach, a relatively simple and straightforward method, can dramatically reduce the wrapper method’s running time with little or no accuracy loss, and in some case achieve higher accuracies than those yielded by wrapper-selected sets-another example of applying Occam’s razor in machine learning, suggesting the simplest hypothesis is the best (Mitchell, 1997). Analysis of experimental results also reveals that the hybrid approach can achieve the goal of maximum relevance with minimum redundancy.

In this paper, we first briefly introduced different gene selection methods evaluated in our empirical study, then the hybrid gene selection approach for biomarker discovery was proposed.

## Gene selection methods

This paper evaluates six filter methods and three wrapper methods. In this section, we briefly introduced these methods.

### Filter methods

*Fisher’s ratio* (*FR*) Fisher’s ratio is a measure for (linear) discriminative power of some variable, and it is defined as:

(1) $$FR=\frac{{\left(m_{1}-m_{2}\right)}^{2}}{\left(v_{1}+v_{2}\right)}$$

where m_1_ and m_2_ are the means of the expression level of a particular gene in class 1 and class 2; *v*_1_ and *v*_2_ are the corresponding variances. In our experiments, given a microarray dataset, we compute the FR value for each gene and rank them in descending order. Genes with higher ranking have more discriminative power for classifying samples into categories.

*Information gain* (*IG*) Information gain is a measure of the effectiveness of an attribute in classifying the training data. It is based on entropy, a measure commonly used in information theory (Mitchell, 1997). Information Gain *IG*(*S,A*) measures the number of bits saved (information obtained) when encoding the target value of an arbitrary member of *S*, by knowing the value of attribute *A*. In our case, given a microarray dataset, we compute the information gain for each gene and rank them in descending order by their information gain value. The higher information value a gene has, the more effective the gene used to classify the training data.

*ReliefF* While gene selection using measures such as Fisher’s ratio and Information gain assume the conditional independence of the attributes (in our case, genes), Relief algorithms (Relief, ReliefF and RReliefF) consider the dependencies between attributes (Robnik-Sikonja and Kononenko, 2003). The basic idea of Relief algorithm is to measure how well attributes distinguish between samples that are near to each other. Original Relief is limited to two-class classification. ReliefF extends Relief to solve multi-class classification problems.

*F-test Correlation Difference* (*FCD*) FCD, a minimum redundancy-maximum relevance (MRMR) approach for gene selection, was proposed (Ding and Peng, 2003) to solve the deficiency of simple ranking approaches, which ignore the correlated genes. One key goal of MRMR approach is to require that members in the selected gene sets are maximally dissimilar to each other. Several measures are proposed to achieve this goal, including maximizing features’ Euclidean distances, or minimizing their pairwise correlations. F-test correlation difference (FCD) is one of the proposed criterion functions to achieve minimum redundancy-maximum relevance. F-statistic can be chosen to evaluate relevance between the gene and the class labels (Ding and Peng, 2003; Dudoit et al. 2002). The minimum redundancy condition can be evaluated by Pearson correlation coefficient. If we use V_F_ and W_C_ to represent F-statistic and Pearson correlation coefficient respectively, FCD can be defined as max (V_F_ –W_C_) (Ding and Peng, 2003). During the gene selection process, FCD serves as a criterion to choose genes with minimum redundancy-maximum relevance.

*Generalized Matrix Approximations* (*GMA*) Recently, we proposed a Generalized Matrix Approximation filter method to simultaneously rank the genes and samples to select top k genes for classifying cancer samples (Li et al. 2006). GMA method is based on a resonance model for approximating matrix. It comprehensively considers the global between-class data distribution and local within-class data distribution. By reordering the gene expression data matrix, the expression data distribution can be visually observed. Top ranked genes are differentially expressed across classes and top samples are important to the class (Li et al. 2006).

*Redundancy Based Filter* (*RBF*) *algorithm* Redundancy based filter algorithm is another filter method aimed to select a minimum gene subset with optimum feature relevance and reduced redundancy (Yu and Liu, 2004). The RBF method does not require any threshold for gene relevance or redundancy determination, and outputs the appropriate set of genes which are relevant and not redundant. Furthermore, it reduces the number of feature pairs to be evaluated by combining sequential forward selection with elimination (Yu and Liu, 2004).

### Wrapper methods

In wrapper methods, a classifier is embedded (wrapped) in the feature selection methods. The typical wrapper algorithm searches for feature subsets, evaluates them with the embedded classifier and uses the resulted accuracy as its measure for gene selection (Blum and Langley, 1997). Different classifier and search method combinations can be used for wrapper algorithms. In our experiment, three popular classifiers (briefly introduced below) are chosen, including Naïve Bayes, decision tree and Support Vector Machine. An exhaustive search of the feature space is intractable for such huge dataset as microarray gene expression data. With a cost of little accuracy loss, in this study, we chose a more practical greedy method called greedy stepwise forward search to traverse feature space. This method performs a greedy forward search through the feature subset space, starts with no feature and stops when the addition of any remaining features results in a decrease in evaluation.

### A hybrid approach

Two main objectives in gene selections are: to identify relevant genes for subsequent research and to identify a small set of genes with minimum redundancy, which is to discover biomarkers (Diaz-Uriarte and Alvarex de Andrez, 2006; Li et al. 2006). In order to approach these two objectives, we propose a two-step hybrid gene selection for biomarker discovery, in which, we use the feature estimation from the filter step as the heuristic information for the wrapper step. In the first step, a filter gene selection method is employed to eliminate the irrelevant genes and form a reduced set of genes, and then a wrapper method is applied to the reduced set of genes to find a small set of genes with minimum redundancy. This hybrid approach takes advantages of both filter methods’ efficiency and wrapper methods’ high accuracy. The aim is to find an optimum balance between the precision of the analysis and the computational time. Our gene selection method comparative study showed that Fisher’s ratio, a relatively simple and straightforward method, can achieve similar or even better classification accuracy than other filter methods (see Section 6.1, Results and Discussion). Therefore, in this paper, the Fisher’s ratio is used in the first step to filter out most of the irrelevant features.

### Classifiers

After the gene selection, three state-of-the-art classifiers, decision tree, naïve bayes, and support vector machine, were applied to evaluate the effectiveness of the gene selection methods.

#### Decision tree

We used J4.8 decision tree, which is a Weka implementation of a C4.5 decision tree variant (Witten and Frank, 2005). C4.5 in turn is an extension of the basic ID3 algorithm to avoid overfitting the data, reduce error pruning, handling continuous attributes, improving computational efficiency and other problems.

#### Naïve Bayes

Naïve Bayes (NB) is a statistical learner based on Bayes rules. It is among the most practical approaches to certain types of learning problems (Liu, 2004). Naïve Bayes classifiers assume that any two feature values on a given class label are independent of each other and thus considered to be ‘naïve’.

#### Support Vector Machine

Support vector machine (SVM) is a new generation learning system based on recent advances in statistical learning theory. An SVM classifier creates a hyper-plane that separates the data into two classes as widely as possible. If no linear separation is possible, a non-liner kernel can be employed to transform the data from linear feature space to a non-linear feature space (Li et al. 2004). The training of an SVM classifier is slow compared to Naïve Bayes and Decision Trees and it is not always easy to select the optimal kernel parameters when there is no linear separation is possible.

### Datasets and Experimental setup

Four widely used microarray gene expression data sets are chosen for our experiments: ALL-AML leukemia, lung cancer, breast cancer, and colon tumor. The data is taken from http://sdmc.lit.org.sg/GEDatasets/Datasets.html. Table 1 summarizes these datasets.

We conducted the experiments on these four data sets and compared six filter methods and three wrapper methods with NB, J4.8 and SVM being the embedded classifier respectively. We also evaluated the hybrid approach we proposed. We used Weka, a well known comprehensive toolset for machine learning and data mining (Witten and Frank, 2005), as our main experimental platform. For the filter methods, we used Weka implementation of Information Gain and ReliefF. RBF was implemented in the Weka environment by Yu’s team (Yu and Liu, 2004). Fisher’s Ratio, GMA and FCD methods were implemented with MATLAB, a high-performance language for technical computing. We evaluated the performance of different filter and wrapper methods in Weka environment with three classifiers, NB, J4.8 and SVM, using Leave-One-Out Cross Validation (LOOCV). We performed LOOCV on both the feature selection process and the classification step. We conducted our experiments on a Pentium IV machine with 2G RAM. Weka (3.4.6) and MATLAB2006a software packages were used. Linear kernel was applied when SVM was used as the classifier.

## Results and discussion

### Filter methods

For each filter method, except RBF method, the genes were ranked based on the scores the feature selection methods assigned. Only the top-ranked genes were selected for classification purpose. The number of top-ranked genes (k) tested were k = 2, 4, 10, 20, 50, 100, 200, 500, 1000. An exception is RBF method, which outputs a fixed number of genes for each data set. Therefore, no top k genes can be selected to test. For comparison, LOOCV percentage accuracy for the full gene set without selection was also evaluated for each dataset. LOOCV was applied to validate each classifier on different gene sets selected by different filter methods.

**Figure 1:**
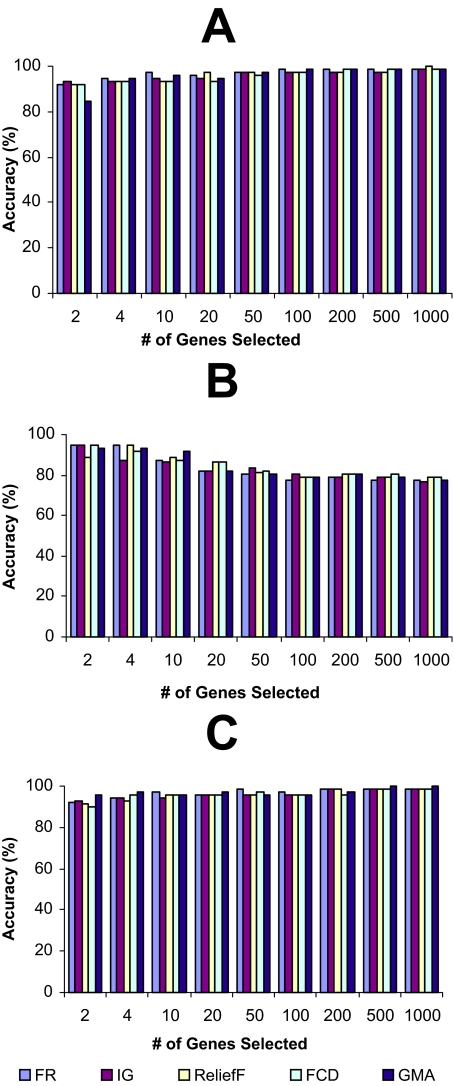
The leave-one-out-cross-validation accuracies of leukemia dataset. The genes were ranked by different filter methods and top-ranked k genes were selected for a classifier to classify the samples. (A): Support Vector Machine (SVM); (B): Decision tree J4.8; (C): Naïve Bayes (NB).

**Figure 2:**
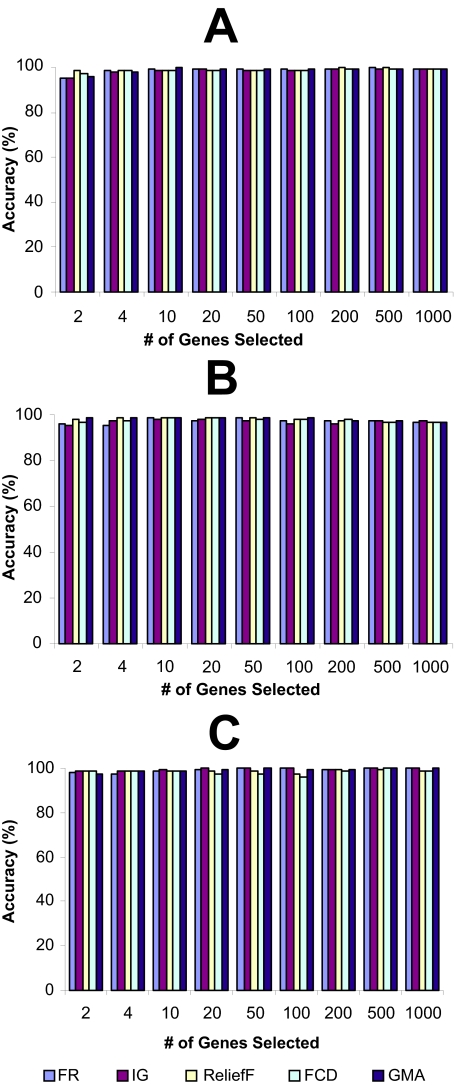
The leave-one-out-cross-validation accuracies of lung cancer dataset. The genes were ranked by different filter methods and top-ranked k genes were selected for a classifier to classify the samples. (A): Support Vector Machine (SVM); (B): Decision tree J4.8; (C): Naïve Bayes (NB).

**Figure 3:**
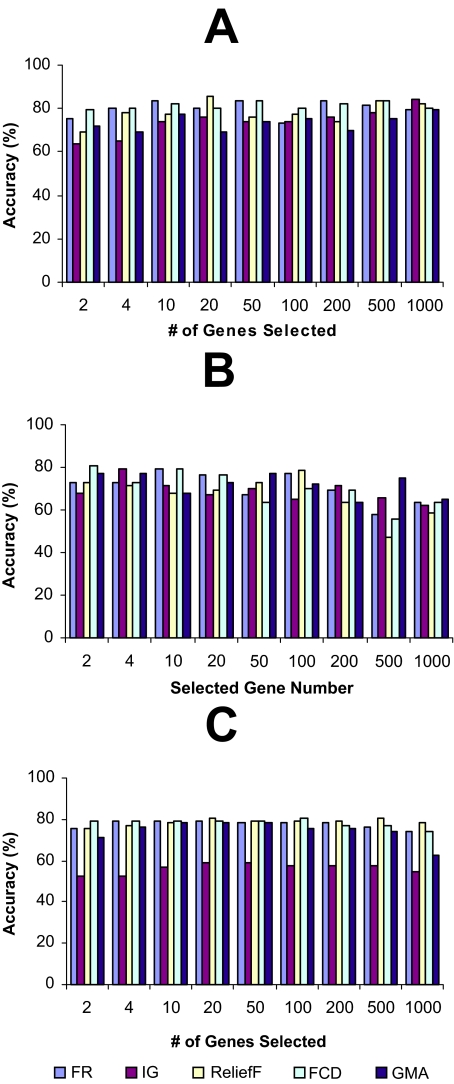
The leave-one-out-cross-validation accuracies of breast cancer dataset. The genes were ranked by different filter methods and top-ranked k genes were selected for a classifier to classify the samples. (A): Support Vector Machine (SVM); (B): Decision tree J4.8; (C): Naïve Bayes (NB).

**Figure 4:**
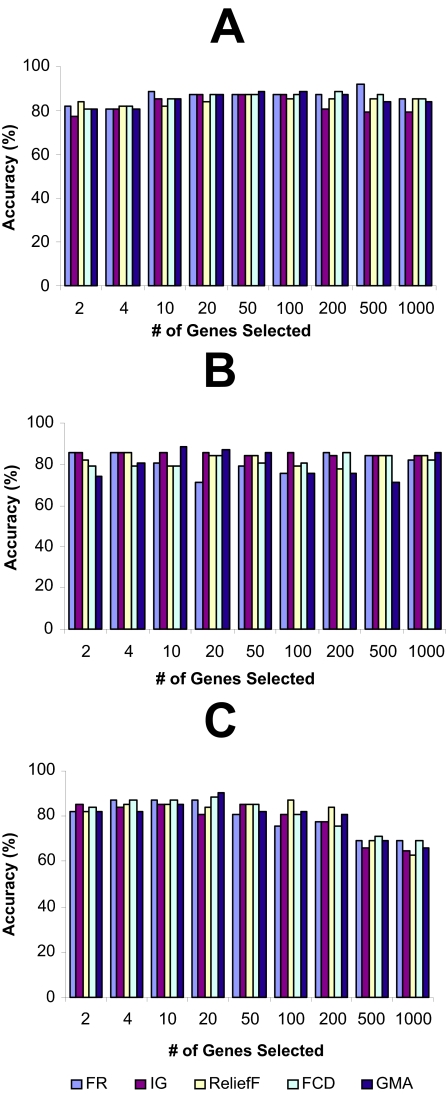
The leave-one-out-cross-validation accuracies of colon cancer dataset. The genes were ranked by different filter methods and top-ranked k genes were selected for a classifier to classify the samples. (A): Support Vector Machine (SVM); (B): Decision tree J4.8; (C): Naïve Bayes (NB).

The LOOCV accuracies, achieved using different top-k-ranked genes selected from the four microarray data sets by FR, IG, ReliefF, FCD, and GMA, were shown in Figures 1–4, while the LOOCV accuracies, achieved using the genes selected by RBF were shown in Table 2, Table 3 showed the LOOCV accuracies when all the genes in each microarray data sets were used without gene selection for classification purpose. From the results on leukemia and lung cancer datasets (Figures 1 and 2, Table 2), we can see that all six filter methods have high LOOCV accuracies and perform almost equally well. Results on breast cancer and colon tumor datasets (Figures 3 and 4, Table 2) showed significantly lower accuracy for all tested filter methods than leukemia and lung cancer datasets, indicating that these two datasets are noisier than lung cancer and leukemia data sets. RBF performs better when J4.8 is used as the classifier, but has lower accuracy when SVM or NB was used as the classifier, especially when it was tested on breast and colon cancer data sets. The results also showed that RBF method selected 67 genes out of 24481 genes in breast cancer dataset while some other methods achieved higher accuracy by selecting less number of top-ranked genes. Yu and Liu (2004) claimed that RBF is an efficient method to discover subset of genes with maximum relevance and minimum redundancy. The fact that RBF selected more genes (67 genes in Breast cancer dataset) than other methods while achieved lower accuracies showed that at least in breast cancer dataset, RBF is not so effective as other filter methods in biomarker discovery. Another interesting point is that information gain (IG) filter method performed significantly worse than other methods when NB is applied as the classifier and tested on the two noisy datasets, breast cancer and colon cancer. Other than these, there is no single filter method that performs universally better than others. It is difficult to select the best gene selection method because no clear winner seems to exist (Li et al. 2004). Not all machine learning methods are created equal. Knowing which method works the best for a given problem is not inherently obvious(Cruz and Wishart, 2006). However, the experimental results reveal that despite its simplicity, the Fisher’s ratio, a traditional statistical method, performed at least as well as or even better than some newly developed complicated gene selection methods in most of the cases (Figures 1–4, and Table 2).

### Wrapper methods

Table 4 presents the summaries of the running time, LOOCV accuracy rate of wrapper methods using NB, J4.8 and SVM as the embedded classifier respectively. The results showed that wrapper methods have significantly higher accuracy than filter methods, especially for those “noisy” datasets (breast cancer and colon tumor datasets) (Table 4, Figures 3 and 4, and Table 2). Wrapper methods’ better accuracy comes with the cost of computational complexity. As the results showed, wrapper methods are more time consuming than the filtering methods. Among the three wrapper methods using different embedded classifiers, the one with SVM embedded is the most time-consuming without significantly better accuracy.

### Hybrid approach: FR-Wrapper

In order to take advantage of both filter methods’ efficiency and wrapper methods’ high accuracy, we propose a hybrid approach by running wrapper methods over a gene subset pre-selected by a filter method. In these experiments we selected Fisher’s ratio as the pre-selecting filter method. Fisher’s ratio was chosen to perform the pre-selection process due to its simplicity, computational efficiency and performance consistence over the four tested gene expression datasets, as analyzed in section 6.1. In order to test if the hybrid approach can achieve improvement over “pure” filter and “pure” wrapper methods, we conducted the experiments on the four microarray datasets. Table 5 showed the experimental results of our hybrid approach tested on the four datasets. The gene numbers in the second column of Table 5 are the numbers of genes preselected by the filter method, Fisher’s ratio, before wrapper methods were applied. In our study, we preselected 200, 100 and 50 top-ranked genes using Fisher’s ratio method. Then the wrapper methods were employed to select biomarker on the search space of 200, 100 and 50 genes.

From the results we have the following observations:

1. The hybrid approach achieved the same accuracies as or even higher accuracies than the wrapper methods when leukemia and lung cancer datasets were analyzed. When leukemia data was analyzed, the hybrid approach with NB wrapper method (accuracy of 100%) (Table 5) outperforms simple NB wrapper method (accuracy of 98.61%) (Table 4). The hybrid approach runs much faster than the simple wrapper methods (The running time reported here includes both the gene preselection by Fisher’s ratio stage and the wrapper gene selection stage). When the hybrid approach was tested on the leukemia dataset, it took the hybrid approach with NB wrapper method about 6 seconds to finish the test with 100% accuracy (Table 5), while it took the simple NB wrapper method about 240 seconds to finish the test with 98.61% accuracy (Table 4). More significantly running time reduction can be observed when SVM wrapper was tested with gene pre-selection by Fisher’s ratio (hybrid approach, 566 seconds with 50 pre-selected genes) (Table 5) or without gene pre-selection (simple SVM wrapper method, 55980 seconds) (Table 4). When the two “noisy” datasets, breast cancer and colon cancer datasets were tested, the hybrid approach is more computational efficient with small accuracy lost.
2. The hybrid approach significantly outperforms the simple filter method with higher classification accuracies. When breast cancer dataset was tested, the hybrid approach can achieve 88.66% accuracy with SVM wrapper method and 6 genes were selected as biomarker (Table 5), while Fisher’s ratio filter method alone achieved 80.41% accuracy when 4 genes were selected, and 83.51% accuracy when 10 genes were selected (Fig. 3). Similar trends can be observed when other three microarray datasets were tested.
3. There is almost no accuracy loss when the number of pre-selected genes was reduced from 200 to 50. In some cases, even higher accuracies were achieved. Therefore, for the four microarray datasets tested in this paper, in order to achieve reasonable accuracy and computational efficiency, a hybrid approach, which combines Fisher’s ratio filter method to pre-select 50 genes and a wrapper method, is a good candidate for classification purpose.
4. The high accuracy of wrapper methods without pre-selection can be due to overfitting (Das, 2001; Hall, 1999; Kohavi, 1995). In the wrapper method, a search for an optimal feature subset is made using the induction algorithm as a black box. One problem with wrapper method is that of overfitting: the accuracy estimation (such as cross-validation) guides the search toward feature subset that will be good for the specific cross-validation folds, however, overusing the estimate can lead to overfitting (Das, 2001; Hall, 1999; Kohavi, 1995). The hybrid approach chooses more representative gene sets by first filtering out irrelevant genes (to achieve maximum relevance) and then running wrapper methods over the resulting subset (to achieve minimum redundancy). With these advantages, the hybrid approach can effectively be applied in biomarker discovery, the search of a minimal subset of genes that is not only differentially expressed across different sample classes, but also contains most relevant genes without redundancy.

## Conclusions and Future Work

In this work, we evaluated different gene selection methods for biomarker discovery, including some traditional statistical methods and several newly developed methods aimed to obtain maximum relevance and minimum redundancy. Despite their simplicity, the traditional statistical methods such as fisher’s ratio perform at least as well as some newly developed gene selection methods in most of the tested cases. Several wrapper methods are also evaluated. The wrappers methods achieved higher classification accuracy than the filter methods. However, they are biased towards the specific classifier they used to evaluate the alternative subsets, and the high accuracy of wrapper methods may be due to overfitting. We proposed a hybrid approach which combines filter and wrapper methods, in which we use the feature estimation from the filter step as the heuristic information for the wrapper step. In the first step, a filter gene selection method is employed to eliminate the irrelevant genes and form a reduced set of genes, and then a wrapper method is applied to the reduced set of genes to find a small set of genes with minimum redundancy. Our gene selection method comparative study showed that Fisher’s ratio, a relatively simple and straightforward method, can achieve similar or even better classification accuracy than other filter methods. Therefore, in this paper, the Fisher’s ratio is used as the first step to filter out most of the irrelevant features. Furthermore, the hybrid approach can reduce the effect of the overfitting problem and achieve the goal of maximum relevance with minimum redundancy. With these advantages, the hybrid approach may be a good candidate for biomarker discovery from microarray datasets.

One of the future research directions is to analyze the biological meaning of the discovered biomarkers. The consistency of the biomarkers discovered by different methods will also be analyzed.

## Figures and Tables

**Table 1:** Four microarray datasets* we used in this paper.

Dataset	# of genes	# of positive samples	# of negative samples
Leukemia	7129	47 (ALL)	25 (AML)
Lung Cancer	12533	331 (MPM)	150 (ADCA)
Breast Cancer	24481	46	51
Colon Cancer	2000	22	40

* Data was obtained from http://sdmc.lit.org.sg/GEDatasets/Datasets.html

**Table 2:** Classification accuracies of different microarray datasets by RBF.

Data sets	classifier	# of genes selected	Accuracy (%)
Leukemia	NB	4	94.44
	J4.8	4	87.50
	SVM	4	93.06
Lung cancer	NB	6	98.90
	J4.8	6	98.34
	SVM	6	96.13
Breast cancer	NB	67	61.85
	J4.8	67	79.38
	SVM	67	75.26
Colon cancer	NB	4	77.42
	J4.8	4	93.55
	SVM	4	80.65

**Table 3:** Classification accuracies of different microarray datasets without gene selection.

Data sets	# of genes	classifier	Accuracy (%)
Leukemia	7129	NB	100
		J4.8	73.61
		SVM	98.61
Lung cancer	12533	NB	97.79
		J4.8	96.13
		SVM	99.45
Breast cancer	24481	NB	52.57
		J4.8	52.58
		SVM	69.07
Colon cancer	2000	NB	58.64
		J4.8	80.65
		SVM	82.26

**Table 4:** Classification accuracies of different microarray datasets by three wrapper methods (SVM-forward selection, NB-forward selection, and decision tree J4.8-forward selection).

Data sets	classifier	time (seconds)	# of gene selected	Accuracy (%)
Leukemia	NB	360	3	98.61
	J4.8	360	2	95.83
	SVM	55980	5	98.61
Lung cancer	NB	1080	3	100
	J4.8	1560	2	99.45
	SVM	59760	4	100
Breast cancer	NB	5280	3	88.66
	J4.8	13920	2	93.81
	SVM	447060	4	89.69
Colon cancer	NB	300	8	93.55
	J4.8	300	3	96.77
	SVM	12060	5	91.94

**Table 5:** Classification accuracies of different microarray datasets by the hybrid approach.

Datasets	Search space	Classifier	time (seconds)	# of gene selected	Accuracy (%)
Leukemia	200	NB	13	4	100
	200	J4.8	13	2	95.83
	200	SVM	896	3	98.61
	100	NB	7	4	100
	100	J4.8	7	2	95.83
	100	SVM	566	4	98.61
	50	NB	4	4	100
	50	J4.8	4	2	95.83
	50	SVM	273	4	98.61
Lung cancer	200	NB	16	3	100
	200	J4.8	30	2	99.45
	200	SVM	652	3	100
	100	NB	8	3	100
	100	J4.8	16	2	99.45
	100	SVM	327	3	100
	50	NB	4	3	100
	50	J4.8	8	2	99.45
	50	SVM	157	3	100
Breast cancer	200	NB	34	6	84.54
	200	J4.8	84	4	86.60
	200	SVM	1032	3	82.47
	100	NB	13	5	86.60
	100	J4.8	42	4	85.57
	100	SVM	886	6	88.66
	50	NB	7	5	86.60
	50	J4.8	15	3	85.57
	50	SVM	427	6	88.66
Colon cancer	200	NB	12	5	91.94
	200	J4.8	36	3	96.77
	200	SVM	1079	5	90.32
	100	NB	6	5	91.94
	100	J4.8	16	3	90.32
	100	SVM	539	5	90.32
	50	NB	4	5	90.32
	50	J4.8	8	3	90.32
	50	SVM	246	4	87.09
